# Effects of forest composition and structure on breeding bird communities in Mount Edough (north-eastern Algeria)

**DOI:** 10.3897/BDJ.13.e172987

**Published:** 2025-12-01

**Authors:** Zahra Brahmia, Rahma Machtoub, Slim Benyacoub, Nadia Ziane, Rachid Rouag

**Affiliations:** 1 Department of Biology, Faculty of Science, Chadli Bendjedid University, El Tarf, Algeria Department of Biology, Faculty of Science, Chadli Bendjedid University El Tarf Algeria; 2 Department of Biology, Faculty of Sciences, Badji Mokhtar University, Annaba, Algeria Department of Biology, Faculty of Sciences, Badji Mokhtar University Annaba Algeria; 3 1Laboratory of Environmental Bio-surveillance, Department of Biology, Faculty of Sciences, Badji Mokhtar - Annaba University. 12, P.O.BOX. 2300, Annaba, Algeria 1Laboratory of Environmental Bio-surveillance, Department of Biology, Faculty of Sciences, Badji Mokhtar - Annaba University. 12, P.O.BOX. 2300 Annaba Algeria; 4 Laboratory of Environmental Sciences and Agroecology, Department of Agronomy, Faculty of Sciences, Chadli Bendjedid University, El Tarf, Algeria Laboratory of Environmental Sciences and Agroecology, Department of Agronomy, Faculty of Sciences, Chadli Bendjedid University El Tarf Algeria

**Keywords:** birds, assemblage, forest, diversity, Edough, Algeria

## Abstract

This study examines the effects of forest vegetation structure on the richness, abundance and composition of bird communities in four different Mediterranean habitats in north-eastern Algeria. Thirty bird species were recorded in the four sampled habitats, belonging to six orders and 14 families. The order Passeriformes is the most represented, with 21 species. Results indicate that cork oak (*Quercus suber*) and zeen oak (*Quercus faginea*) forests, characterised by a complex vertical structure and a closed canopy, support a rich and typically forest-dwelling bird community. In contrast, pine (*Pinus pinaster*) forests exhibit lower species richness and are dominated by generalist species, tolerant to habitat disturbance. Vegetation parameters such as tree height (r = 0.56), shrub cover (r = 0.49) and ecological vegetation volume (r = 0.58) were positively correlated with avian richness. With average species richness values of 18.5 and 16.3 species per plot, respectively, zeen oak forests and mixed forests had the highest levels of avian diversity, while pine forests had the lowest (average = 9.7 species).

## Introduction

Forest birds are widely recognised as reliable indicators of ecosystem integrity and environmental quality. Their sensitivity to variations in habitat structure, resource availability and human-induced disturbances makes them valuable tools for assessing the ecological condition of forest environments (Gregory et al. 2005; Devictor et al. 2008). Many ecological studies have highlighted the significant influence of vegetation structure and composition on biodiversity patterns (Ferry and Frochot 1990; Benyacoub 1993; Blondel 1995). Avian communities exhibit strong habitat specificity; the complexity and composition of vegetation significantly affect their diversity and spatial organisation (Caziani and Derlindati 2000; Brawn et al. 2001). This study aims to investigate the spatial distribution patterns of breeding bird communities in the high-altitude forests of the Edough massif across four distinct vegetation structures. Specifically, we seek to assess the richness, composition and structure of breeding avian communities within these habitats. Bird distribution in mountainous regions is particularly complex due to the wide variety of biotopes concentrated within small areas and pronounced altitudinal gradients (Dejonghe 1984). This region is considered a biodiversity hotspot within the Palaearctic ecozone (Comes 2004) and is also recognised as an area of high endemism for both flora (Hamel et al. 2013) and amphibians (Carranza and Arnold 2004; Veith et al. 2004). In recognition of its ecological value, several initiatives have been initiated with the objective of the classification of the Edough massif as a “Natural Park”, covering an area of 35,432 hectares.

This research is motivated by the scarcity of studies on the avifauna of this massif. Existing studies mainly focus on the biology and distribution of specific species, such as the African Blue Tit (*Cyanistes teneriffae*) and the Eurasian Jay (*Garrulus glandarius*) (Chabi et al. 1995; Chabi et al. 2000; Ziane et al. 2006; Belabed et al. 2018; Sakraoui 2019; Zeghdani et al. 2024). Conversely, few investigations have undertaken a comprehensive inventory of bird species and evaluated the conservation status of the main forest formations of the massif (Zeghdani et al. 2024). The inventory and assessment of bird communities, particularly breeding species, provide reliable indicators of forest ecosystem health. Within this context, the present study aims to enhance current knowledge on the breeding birds of the Edough region and to assess their long-term conservation status. To achieve these objectives, we implemented a methodological approach combining standardised ornithological surveys with an analysis of forest habitat characteristics.

## Materials and Methods

### Study site

This study was conducted in Edough massif, situated in north-eastern Algeria. Edough massif is part of the grand Tell Atlas chain running through northern Maghreb. Maximum elevation is 1008 m at Mont Bouzizi. The whole massif is marked by steep gradient from its peak to sea level and is characterised by an important forest cover of Mediterranean Cork Oak *Quercus suber*, Zeen Oak *Quercus faginea* forests and rich scrubs (Boulahbal et al. 2022). The climate of the region is typically warm Mediterranean, with an important rainfall difference between the mountains and the coast (1200 and 700 mm), respectively. Bird sampling was carried out on the northern slope of the massif, at an altitude between 363 and 510 metres (36°55'38"N 7°41'31"E). Sampling took place in the forest on the 8^th^ km of the Col du Chacal track, which is approximately 5.5 km long and runs along the W16 road between Annaba and the village of Seraïdi. This track crosses a dense forest. The slopes are relatively steep. Facing north, the landscape is carved out by numerous valleys that are home to intermittent torrential streams.

### Vegetation structure

**Figure 1.**
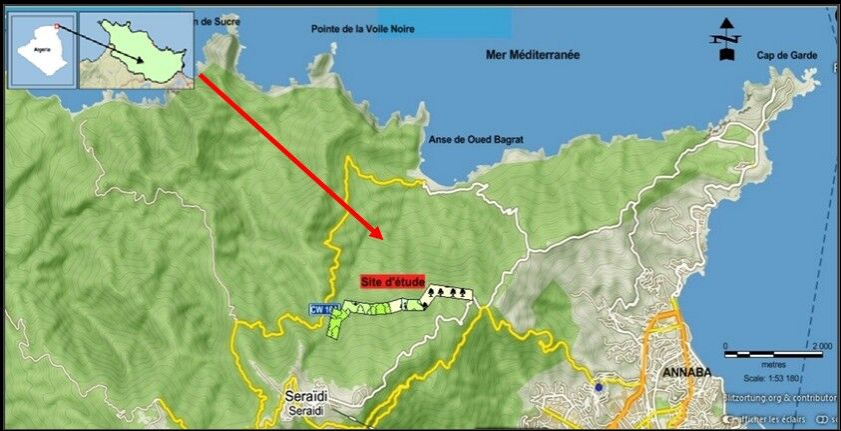
Location of the study site in the Edough massif.

The variation in vegetation structure was used to study the community structure of bird species. Four forest habitats were identified along a 200 m wide, 5 km long sampling transect:

- A pine forest (*Pinus pinaster*) was found to correspond with a rugged terrain formation where bedrock was occasionally visible. The sparse undergrowth is dominated by *Retama sphaerocarpa*, *Calycotome spinosa*, *Erica arborea* and *Cistus monspelliensis*.

- A cork oak forest (*Quercus suber*), whose undergrowth comprises *Calycotome vilosa*, *Erica arborea*, *Cistus monspelliensis*, *Arbutus unedo*, *Viburnum tinus*, *Cytisus triflorus*, *Phillyrea angustifolia* and *Myrtus communis*.

- A mixed formation whose tree layer includes zeen oak (*Quercus faginea*), cork oak (*Quercus suber*) and maritime pine (*Pinus pinaster*), with dense undergrowth consisting of *Calycotome vilosa*, *Erica arborea*, *Cistus monspelliensis* and *Cistus salviifolius*.

- A pure zeen oak (*Quercus faginea*) forest, characterised by trees sometimes exceeding 30 metres in height and relatively dense, tall undergrowth consisting of *Calycotome vilosa*, *Erica arborea*, *Cistus salviifolius*, *Arbutus unedo*, *Viburnum tinus* and *Phillyrea angustifolia*. (Fig. 1).

### Environmental variables

To assess the influence of habitat structure on breeding bird richness, a set of vegetation parameters was measured within each sampling plot. The average height (TLH) and cover (TLC) of trees and the average height (SLH) and cover (SLC) of shrub vegetation were evaluated in every station. The DBH (diameter at breast height) was also calculated. This variable is used to monitor tree growth and calculate both net production and basal area of forest plots. During each bird census, we used a tape measure to measure the diameter at DBH of about ten trees in the station.

An index of Ecological Vegetation Volume (EVV) was then calculated to quantify the vertical complexity of the vegetation. EVV combines the mean height and the cover of each vegetation layer according to the formula: \begin{varwidth}{50in}\begin{equation*}
            EVV = \sum_{i=1}^{n} (H_i \times C_i)
        \end{equation*}\end{varwidth}

- where *Hi* and *Ci* represent the mean height and cover percentage of layer *i*, respectively. This index provides an integrated measure of structural complexity reflecting both vertical and horizontal vegetation development.

### Avifauna sampling

The point count method is a widely used technique for sampling birds across the world due to its effectiveness and easy implementation (Diefenbach et al. 2003). This method allows us to estimate species richness amongst different habitats (Barlow et al. 2007, Castaño-Villa et al. 2014). We estimated species densities using conversion coefficients calculated by Benyacoub (1993). The study site was surveyed weekly from April 2019 to mid-May 2019, for all habitat types. We performed 24-point counts (Suppl. material 1). For each point count, we used a timed count of 20 min in a radius of 10 m. All counts were made by the same person (ZB) to avoid inter-observer bias. Sampling was done mainly during the morning (6:30 to 10:30 am) when birds are found to be most active. Each point was separated by at least 150 m from all other points to minimise the probability of sampling the same bird more than once. We estimated species densities using conversion coefficients calculated (Benyacoub 1993) by converting IPA values to the number of pairs/10 ha to allow comparison between habitats.

### Data Analyses

To examine the bird community composition, we used species richness “S” that is defined as the total number of species found in a given habitat. To compare the composition of breeding bird communities amongst habitats, a Venn diagram was performed to plot common and biotope-specific bird species of each biotope (Heberle et al. 2015). The diversity was calculated using the diversity index of Shannon-Weaver.

Richness was correlated with structural parameters using Pearson’s correlation coefficient (r). Pearson’s correlation coefficients were computed using SPSS software (version 11.0.1). We calculated the asymmetrical Hellinger distance between sampling sites using R 4.5.0. Hellinger distance is a highly recommended distance for analysis, based on abundance data (Legendre and Legendre 2012). To explore the relationship between bird community composition and forest structure, a redundancy analysis (RDA) was performed by using *Statistica (14.0.1*.) software. It allows the direct identification ecological interpretation of species-habitat relationships (ter Braak 1986).

## Results

### Structure of vegetation

The four study stations are presented as a succession of vegetation where the degree of coverage and height evolve more or less positively and regularly from one station to another (Table 1).

The analysis of vegetation structural parameters revealed differences amongst the four forest types studied. Tree layer height (TLH) varied considerably, being lowest in pine forests (10.97 ± 3.71 m) and highest in Zeen oak forests (20.01 ± 3.68 m). Tree layer cover (TLC) followed a similar trend, with higher values in mixed and Zeen oak forests (46.25 ± 12.48% and 45.71 ± 7.50%) and lower in pine forests (35 ± 14.14%). Shrub layer height (SLH) was lowest in pine forests (2.35 ± 0.07 m) and highest in cork and Zeen oak forests (≈ 3.1 m). Conversely, shrub layer cover (SLC) was greatest in pine forests (73.5 ± 9.19%) and decreased in denser forests, especially Zeen oak (52.46 ± 17.14%). Mean tree diameter at breast height (DBH) ranged from 43.91 ± 2.12 cm in cork oak to 56.94 ± 12.26 cm in Zeen oak forests. The Ecological Vegetation Volume (EVV) increased steadily from pine (414.13 ± 13.97) to Zeen oak forests (1328.92 ± 294.38).

### Birds diversity

The avifaunal composition consists of 30 bird species spread across six orders and 14 families. The order Passeriformes is by far the most represented, with 21 species accounting for 70% of the total. Four orders have only one species each: Piciformes (three species; 10%), Columbiformes (two species; 6.67%) and Accipitriformes (two species; 6.67%). Coraciiformes and Galliformes were the least represented taxa, accounting for just 3.33% of the total diversity. Fringillidae is the most species-rich family with six species (20%). Paridae, Muscicapidae and Picidae follow with three species each (10%). The other families are represented by one or two species each. According to their phenological status, 3.33% of the birds were unintentional visitors, 6.67% were winter visitors, 6.67% were passage migrants and 63.33% were resident breeders. According to the IUCN Red List, all of species (100.00%) were categorised as least concern. The mixed formation (26.25%) and the Pine forest (13.75%) had the lowest richness and were followed by the Cork oak forest and Zeen oak forest, which had 30.00% and 30.00% of habitat-specific records, respectively. The Zeen oak forest was the richest habitat, with 24 bird species (30.00% of all occurrences) (Table 2).

### Species distribution across habitats

**Figure 2.**
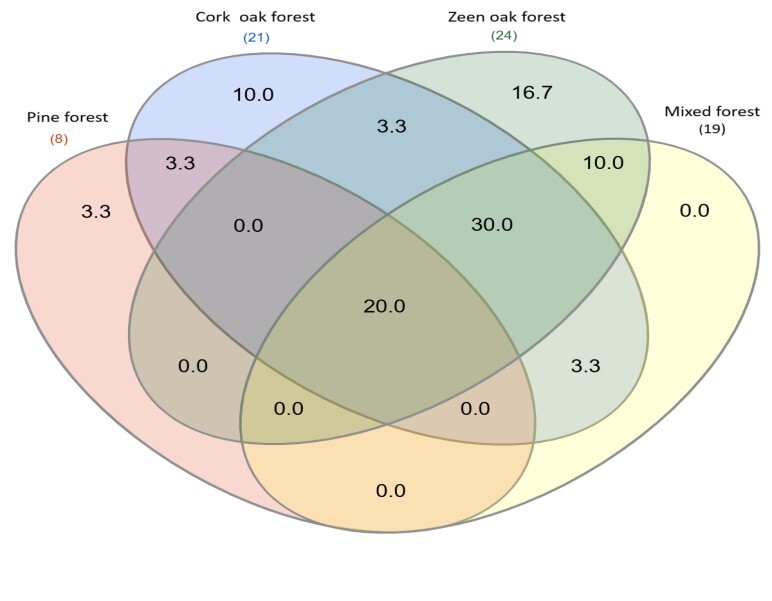
Venn diagram of bird species percentage occurring over the four habitats.

The Venn diagram below shows the distribution of bird species across four forest habitats. It reveals clear patterns of species overlap. Of the 30 species observed, 30.0% were found in all four habitats. This core group comprises generalist, including the European Robin (*Erithacus rubecula*), the Common Blackbird (*Turdus merula*) and the Great Tit (*Parus major*). Some species are strongly associated with the Cork oak habitat, as evidenced by its 10.0% contribution of unique species, including the Western Bonelli's Warbler (*Phylloscopus bonelli*) and the Orphean Warbler (*Curruca hortensis*). The Common Chaffinch (*Fringilla coelebs*) and the European Greenfinch (*Chloris chloris*), for example, demonstrate wide ecological tolerance by inhabiting all or most habitats (Fig. 2).

### Communities structure

The structural indices of the various forest habitats clearly show a gradient ranging from evergreen formations, represented by cork oak, to deciduous formations, represented by zeen oak. In general, pine forests are the least diverse, with only eight species. There is a steady increase in richness, diversity index and evenness index in the three other climax formations, from the cork oak forest to the zeen oak forest (Table 3).

Forest environments with multiple tree layers have the highest species densities. Deciduous environments have significantly higher densities than evergreen environments. Zeen oak has the highest total density, which is 25% higher than Cork oak and almost three times higher than Pine forest.

**Figure 3.**
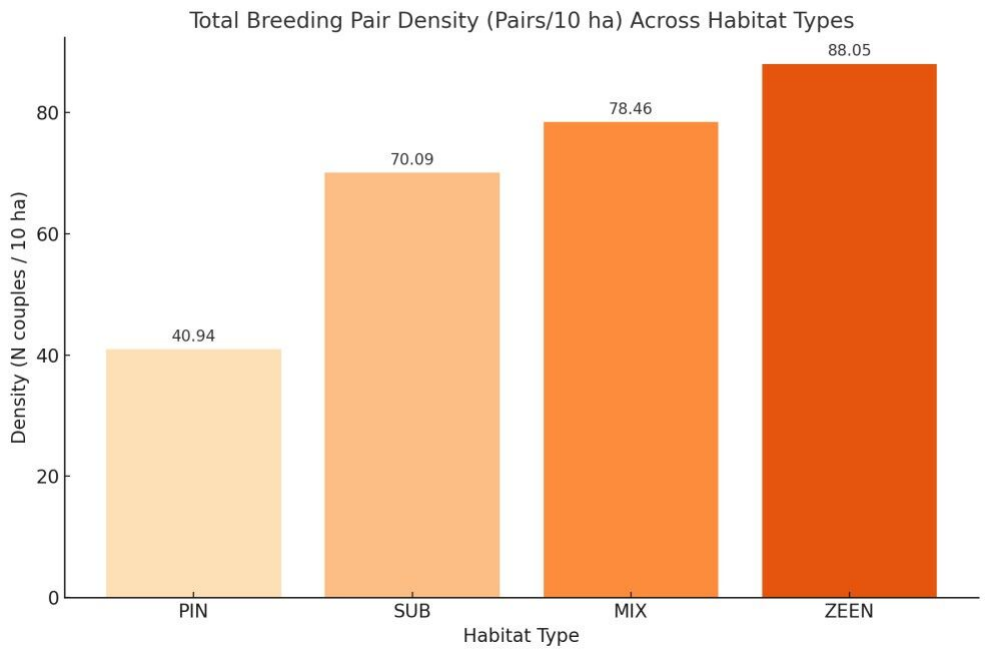
Total breeding density (pairs/10 ha) across habitat types.

The density of birds varied significantly depending on the type of habitat. Zeen oak forest, with its structurally complex vegetation, had the highest total breeding pair density (88.05 pairs/10 hectares). Mixed forest (78.46 pairs per 10 hectares) and (Cork oak 70.09 pairs per 10 hectares) followed. Pine forest had the lowest density (40.94 pairs/10 ha) (Fig. 3).

Clear patterns of species dominance and composition existed within the bird populations of the four forest habitats (measured in pairs per 10 hectares). The European robin (*Erithacus rubecula*) was the most common species in all habitats. In pine forests, eight pairs were found per 10 hectares, whereas in *Quecus suber* environments, this figure was 14.6 pairs per 10 hectares. This demonstrates its ability to adapt to a wide range of environments, indicating a generalist approach. In contrast, the Sardinian warbler (*Sylvia melanocephala*) had a high density in pine forests (19.5 pairs per 10 hectares), suggesting a preference for open or conifer-dominated habitats. Conversely, its low density (0.7 pairs per 10 hectares) in zeen oak areas suggests that it avoids these habitats. Blackcaps (*Sylvia atricapilla*) and blue tits (*Cyanistes teneriffae*) are found in large numbers in many types of habitat, but are most prevalent in zeen oak and cork oak woodlands. This shows that they favour wooded environments with complex structures. Mixed forests with lots of undergrowth also have high densities of chiffchaffs (*Phylloscopus collybita*) and wrens (*Troglodytes troglodytes*). Overall, Pine forests hosted fewer species with high densities, notably the Sardinian Warbler (*Curruca melanocephala)* and the Robin (Erithacus rubecula), while Zeen forests exhibited the highest densities for most species, indicating a richer and more structurally diverse environment.

**Figure 4.**
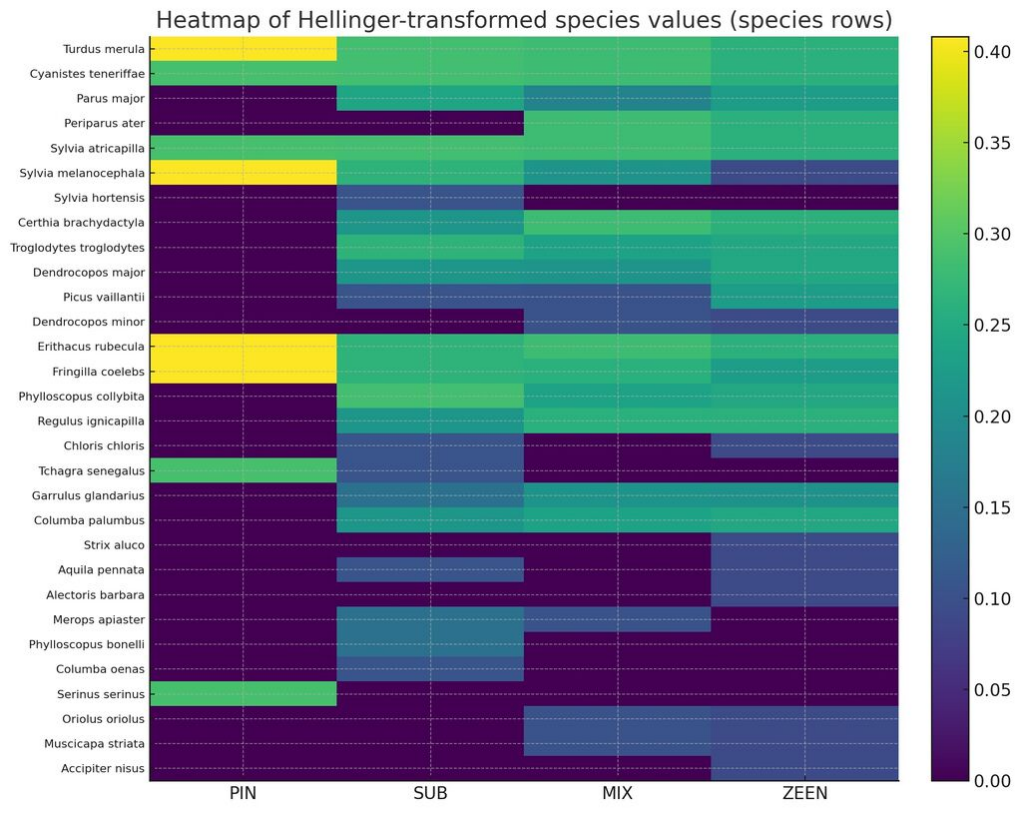
Heatmap with Hellinger-transformed bird abundance values across forest habitat types (PIN, SUB, MIX, ZEEN).

The structure of bird communities was also explored using a heatmap, based on the Hellinger transformation to visualise variations in relative abundance amongst different forest habitats. The heatmap clearly shows differences in species composition across habitats. Mixed forest and Zeen oak forest exhibit the highest relative abundances and species diversity. Generalist species, such as Turdus merula, Cyanistes teneriffae, Sylvia atricapilla and Erithacus rubecula, maintain consistently high abundances across all habitats, indicating ecological plasticity. Conversely, forest-specialist species, such as *Periparus ater*, *Certhia brachydactyla*, *Regulus ignicapilla* and *Columba palumbus*, are more strongly associated with denser, more structurally complex habitats. Pine forest and cork oak forests display lower species richness and are dominated by Fringillidae and Sylviidae taxa, such as *Sylvia melanocephala*, *Fringilla coelebs* and *Serinus serinus*. Overall, the heatmap shows an increasing gradient of species richness and abundance from pine to Zeen oak forests, which is consistent with the progressive growth in forest structural complexity (Fig. 4).

### Vegetation structure and relationships with bird community

**Figure 5.**
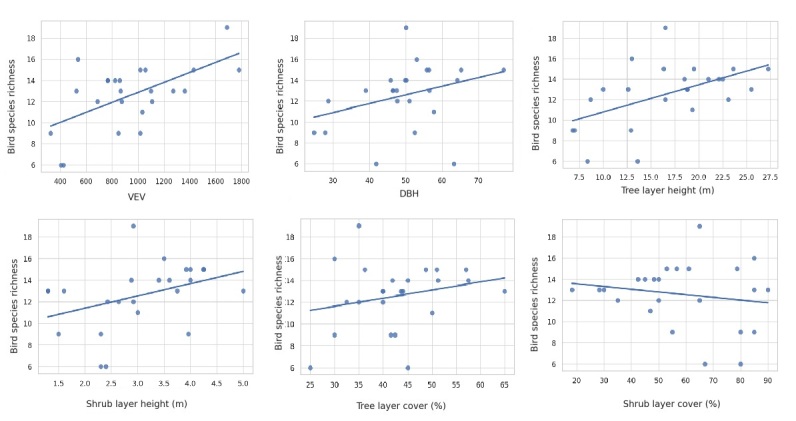
Relationship between vegetation structure and bird species richness.

Our analysis revealed clear patterns linking vegetation structure to bird species richness across the sampled Mediterranean forest habitats. The scatterplots highlighted that vertical structural features of vegetation are strong predictors of avian diversity. The strongest positive correlation was observed for the Ecological Vegetation Volume (EVV) (r = 0.58), a composite index integrating both the height and cover of vegetation layers. Similarly, canopy height was highly correlated with species richness (r = 0.58, p < 0.05). Moderate positive correlations were also detected with shrub height (r = 0.38, p < 0.05) and with tree diameter at breast height (r = 0.32, p > 0.05), though the latter was not statistically significant. In contrast, shrub cover showed a moderate, but non-significant negative relationship with species richness (r = –0.17, p > 0.05), whereas canopy cover exhibited a weak and non-significant positive association (r = 0.23, p > 0.05) (Fig. 5).

**Figure 6.**
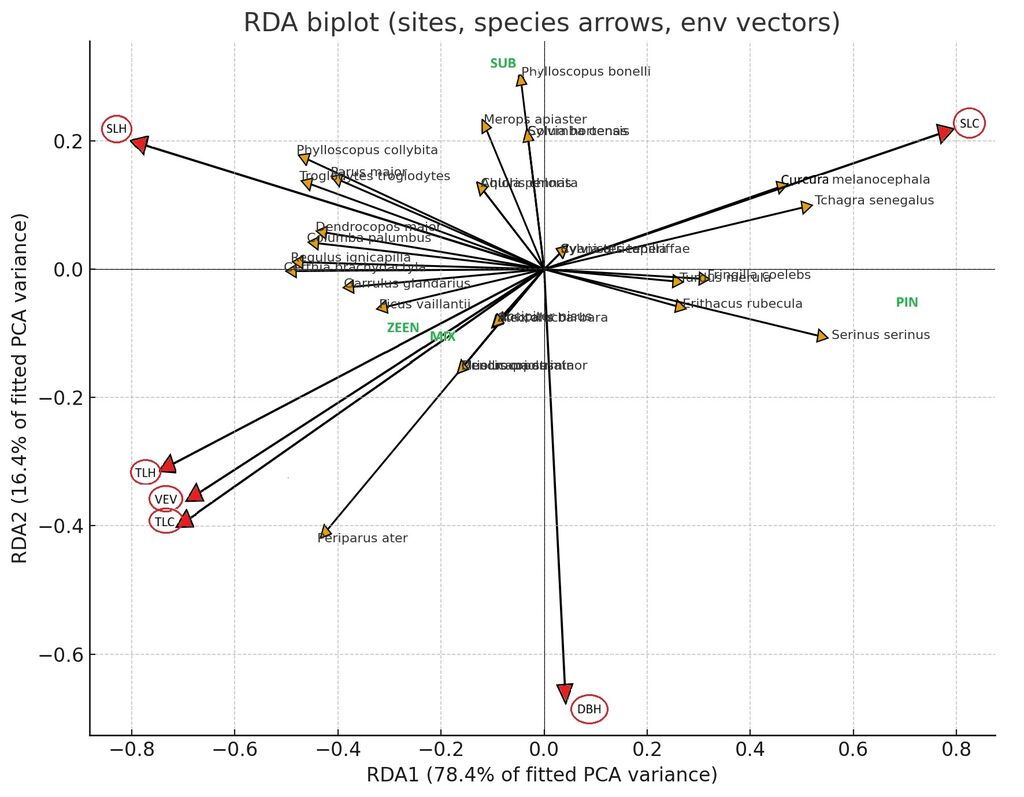
RDA biplot showing relationships between breeding bird species, vegetation structure variables (red circle) and forest habitats (PIN; SUB; MIX and ZEEN) in the Edough massif.

The redundancy analysis (RDA) revealed a clear separation of bird communities according to forest structure and habitat type. The first two RDA axes together explained 94.8% of the constrained variance (RDA1 = 78.4%, RDA2 = 16.4%). RDA1, which accounted for most of the explained variance, represented a major ecological gradient from open pine to dense oak forest. On the positive side of RDA1, sites characterised by a low canopy cover and a high percentage of shrub cover were associated with open habitat and xerophilous species, such as *Sylvia melanocephala*, *Tchagra senegalus* and *Serinus serinus*, mainly recorded in the Pine forest (PIN). Conversely, the negative side of RDA1 was correlated with high values of tree cover (TLC), tree height (TLH) and Diameter at Breast Height (DBH), reflecting mature and closed canopy forests. This part of the gradient corresponded to the Zeen and mixed forests supporting forest specialist birds, such as *Parus ater*, *Dryobates minor*, *Garrulus glandarius*, *Picus vaillantii* and *Regulus ignicapilla*. RDA2 (16.4% of the constrained variance) and expressed a secondary gradient related to intermediate vegetation structures and Shrub composition. The upper part of this axis was associated with the cork oak forest (SUB), which supported semi-open woodland species such as *Phylloscopus bonelli*, *Merops apiaster* and *Sylvia borin*. The lower part of RDA2 corresponded to the Zeen forest (Zeen), characterized by dense canopy and mature forest birds (*Parus ater*) (Fig. 6).

## Discussion

In this study, we show that bird richness and species occurrence frequency vary depending on the structure of the vegetation in the mountain area. An increase in species richness along a gradient of plant structure complexity has been observed in north-eastern Algeria and in European forests, the richness of bird species being observed in the most advanced stages (Blondel et al. 1973; Müller 1985; Rocamora 1987; Benyacoub 1993; Laiolo et al. 2004) then, habitat structure affects the composition and diversity of forest bird communities. In terms of tree species composition, most studies in temperate forests have focused on the difference between deciduous and coniferous forests (Donald et al. 1998; Easton and Martin 1998; Díaz 2006; Reif et al. 2008). More bird species inhabit deciduous rather than coniferous forests (James and Wamer 1982; Willson and Comet 1996; Reif et al. 2008) and elsewhere in the Mediterranean Basin. In our study, results show that structurally complex habitats, such as the Zeen oak forest and the mixed formations, supported the highest diversity and density of bird species, while the pine habitat harboured the lowest values. Several authors found lower species richness in coniferous (James and Wamer 1982; Gil-Tena et al. 2007). Bellatreche (1994) and Moali (1999) demonstrated that woodlands with multi-layered vegetation and a developed shrub stratum exhibit significantly higher bird species richness and densities.

Benyacoub (1993) reported that the species richness and breeding densities of forest birds in the El Kala National Park ranged from 27 to 31 species and from 48.4 to 74 pairs per 10 hectares, respectively, depending on the habitat type. These values are similar to those recorded in the Edough massif. In both regions, Zeen oak forests exhibited the highest bird densities, reflecting their complex vegetation structures and diverse vertical stratification, which provide favourable nesting and feeding opportunities. These similarities suggest that the forest bird assemblages of El Kala and Edough are structurally comparable, reflecting a typical avian diversity pattern of Mediterranean forests in north-eastern Algeria. However, some differences can be noted between the two sites. In the cork oak forest (suberaie) of Edough, density reached 70.09 pairs per 10 hectares, whereas in El Kala, it was lower (48.4 pairs per 10 hectares), likely due to the scarcity of the maquis understorey, which limits the availability of ecological niches for insectivorous passerines. The vegetation structure also differs markedly between the two sites: maquis cover reaches 62.85% in Edough compared to 0.5% in El Kala (Benyacoub, 1993). This structural contrast strongly influences avian diversity and density patterns.

Moreover, our results are in agreement with the work of Bougaham and Moulaï (2014), who emphasised the positive role of vertical vegetation complexity in shaping avian diversity in the Babor Region. Our correlation analyses echo these patterns, revealing that variables, such as Ecological Vegetation Volume (VEV), tree layer height (TLH) and understorey cover (SLC), were the strongest predictors of species richness. In particular, the positive correlations between bird richness and both TLH (r = 0.56) and VEV (r = 0.58) suggest that vertical stratification enhances niche diversity and supports species co-existence. Benyacoub (1993) also reported a strong positive correlation (r = 0.775, p < 0.02) between the vertical vegetation structure (EVV) and the richness of breeding bird communities along a vegetation succession gradient in the El Kala National Park. This suggests that structurally complex habitats offering diverse ecological niches in the vertical space support a higher number of bird species. Ecological Vegetation Volume is positively correlated with the productivity of forest habitats, which explains why its effect on bird communities is the subject of broader consensus (Jokimäki and Huhta 1996).

The distinct bird assemblage found in the Pine forest is indicative of reduced structural complexity and floristic heterogeneity. The analyses further confirm the statistical significance of DBH and SLC in explaining bird richness. Similar findings have been reported by other authors (Barbaro et al. 2005, Gil-Tena et al. 2007) who documented reduced bird diversity in pine forests due to their homogeneity and limited vertical structure. Our results confirmed that bird species composition significantly differs amongst habitats, with clear separation between PIN and ZEEN forests, and the mixed habitat (MIX) acting as an ecological intermediary. Certain bird species emerged as key indicators of specific forest types; *Sylvia melanocephala* was particularly abundant in the PIN habitat, likely due to its preference for sunlit areas with shrub cover (Bas et al. 2010; Papanikolas et al. 2021). Conversely, forest specialists, such as *Periparus ater* and *Dryobates minor* were associated with mature, structurally complex forests like Zeen oak and Mixed forests.

Our study shows the importance of preserving structurally rich and diverse forest habitats in North Africa to maintain avian biodiversity. Forests are complex and more productive habitats, offering a greater diversity of ecological niches (Pautasso and Gaston 2005). Forest management strategies should prioritise the conservation of multilayered vegetation, the presence of deadwood and natural water sources and the maintenance of ecotonal habitats that support a high level of species turnover. These approaches are not only essential for sustaining current avian populations, but also for enhancing the resilience of forest ecosystems in the face of environmental change.

## Conclusions

The Edough massif stands as a true sentinel of Algerian biodiversity, preserving a unique natural heritage where marine, mountainous and forest ecosystems converge. Its rugged topography, humid Mediterranean climate and mosaic of habitats, support a remarkable avifaunal diversity. Numerous Mediterranean forest bird species occur there, whose distribution and abundance are closely linked to the structure and composition of the vegetation. The massif also has a high biogeographical value due to its ecological insularity: surrounded by the sea and urbanised areas, it serves as a refuge for many local animal and plant species. However, this biodiversity is currently threatened by deforestation, recurrent wildfires and, above all, rapid urban expansion in recent years. Being aware of these pressures, the authorities have recently decided to designate Edough as a Natural Park, a measure aimed at slowing degradation, enhancing ecosystem protection and ensuring the long-term preservation of its habitats. This new status should enable the massif to maintain its role as a biodiversity hotspot at the scale of the Mediterranean Basin.

## Supplementary Material

F4785648-49FA-536B-9C29-7EB5BC3F565D 10.3897/BDJ.13.e172987.suppl1 Supplementary material 1 Abundance and density of birds by habitat Data type Abundance File: oo_1414038.xlsx https://binary.pensoft.net/file/1414038 Rouag R.

## Figures and Tables

**Table 1.** Vegetation structure parameters at the four sampled habitats (Mean ± SD). Maritime pine forest (PIN); cork oak forest (SUB); Mixed formation (MIX); zeen oak forest (ZEEN).

	**PIN**	**SUB**	**MIX**	**ZEEN**
**TLH (m)**	10.97±3.71	14.25±6.27	17.2±6.70	20.01±3.68
**TLC (%)**	35±14.14	38.38±5.02	46.25±12.48	45.71±7.50
**SLH (m)**	2.35±0.07	3.14±0.06	3.07±1.26	3.12±1.23
**SLC (%)**	73.5±9.19	62.85±21.57	56.3±24.18	52.46±17.14
**DBH (cm)**	52.59±15.13	43.91±12.12	48.59±12,20	56.94±12.26
**VEV**	414.13±13.97	659.89±206.41	926±146.68	1328.92±294.38

TLH: Tree layer height; TLC: Tree layer cover; SLH: Shrub layer height; SLC: Shrub layer cover; DBH: Diameter at breast height; EVV: Ecological vegetation volume.

**Table 2.** Composition of bird species in the Edough massif by family, habitat, conservation status and phenological status (LC = Least Concern, VU = Vulnerable).

**Familly**	**Species**	**Habitats**	**Conservation Status**	**Phenological Status**
Accipitridae	*Accipiter nisus*	Zeen	LC	Winter visitor
	*Hieraaetus pennatus*	Zeen	LC	Breeding migrant
Phasianidae	*Alectoris barbara*	Zeen	LC	Breeding migrant
Columbidae	*Columba palumbus*	Sub, Mix, Zeen	LC	Resident breeder
	Columba oenas	Sub	VU	Accidental visitor
Picidae	*Dryobates minor*	Mix, Zeen	LC	Resident breeder
	*Picus vaillantii*	Sub, Mix, Zeen	LC	Resident breeder
	*Dendrocopos major*	Sub, Mix, Zeen	LC	Resident breeder
Paridae	*Parus major*	Sub, Mix, Zeen	LC	Resident breeder
	*Periparus ater*	Mix, Zeen	LC	Resident breeder
	*Cyanistes teneriffae*	Sub, Pin, Mix, Zeen	LC	Resident breeder
Certhiidae	*Certhia brachydactyla*	Sub, Mix, Zeen	LC	Resident breeder
Regulidae	*Regulus ignicapilla*	Sub, Mix, Zeen	LC	Resident breeder
Troglodytidae	*Troglodytes troglodytes*	Sub, Mix, Zeen	LC	Resident breeder
Corvidae	*Garrulus glandarius*	Sub, Mix, Zeen	LC	Resident breeder
Oriolidae	*Oriolus oriolus*	Mix, Zeen	LC	Passage migrant
Muscicapidae	*Muscicapa striata*	Zeen	LC	Passage migrant
	*Erithacus rubecula*	Sub, Pin, Mix, Zeen	LC	Resident breeder
Turdidae	*Turdus merula*	Sub, Pin, Mix, Zeen	LC	Resident breeder
Phylloscopidae	*Phylloscopus bonelli*	Sub	LC	Breeding migrant
	*Phylloscopus collybita*	Sub, Mix, Zeen	LC	Breeding migrant
Sylviidae	*Curruca hortensis*	Sub	LC	Breeding migrant
	*Curruca melanocephala*	Sub, Pin, Mix, Zeen	LC	Resident breeder
	*Sylvia atricapilla*	Sub, Pin, Mix, Zeen	LC	Resident breeder
Fringillidae	*Serinus serinus*	Pin	LC	Winter visitor
	*Chloris chloris*	Sub, Zeen	LC	Resident breeder
	*Fringilla coelebs*	Sub, Pin, Mix, Zeen	LC	Resident breeder
Meropidae	*Merops apiaster*	Sub, Mix	LC	Breeding migrant
Strigidae	*Strix aluco*	Zeen	LC	Resident breeder

**Table 3.** Species richness and Shannon diversity and equitability per forest types.

	**PIN**	**SUB**	**MIX**	**ZEEN**
**S**	8	21	19	24
**H**'	2,28	3,44	3,57	3,63
